# Comparison of robotic versus manual needle insertion for CT-guided intervention: prospective randomized trial

**DOI:** 10.1093/radadv/umaf010

**Published:** 2025-04-04

**Authors:** Takao Hiraki, Yusuke Matsui, Jun Sakurai, Koji Tomita, Mayu Uka, Soichiro Kajita, Noriyuki Umakoshi, Toshihiro Iguchi, Michihiro Yoshida, Kota Sakamoto, Takayuki Matsuno, Tetsushi Kamegawa

**Affiliations:** Department of Radiology, Faculty of Medicine, Dentistry and Pharmaceutical Sciences, Okayama University, Okayama 700-8558, Japan; Department of Radiology, Faculty of Medicine, Dentistry and Pharmaceutical Sciences, Okayama University, Okayama 700-8558, Japan; Center for Innovative Clinical Medicine, Okayama University Hospital, Okayama 700-8558, Japan; Department of Radiology, Okayama University Hospital, Okayama 700-8558, Japan; Department of Radiology, Okayama University Hospital, Okayama 700-8558, Japan; Department of Radiology, Okayama University Hospital, Okayama 700-8558, Japan; Department of Radiology, Okayama University Hospital, Okayama 700-8558, Japan; Department of Radiological Technology, Faculty of Health Sciences, Okayama University, Okayama 700-8558, Japan; Center for Innovative Clinical Medicine, Okayama University Hospital, Okayama 700-8558, Japan; Center for Innovative Clinical Medicine, Okayama University Hospital, Okayama 700-8558, Japan; Faculty of Natural Science and Technology, Okayama University, Okayama 700-8530, Japan; Graduate School of Environmental, Life, Natural Science and Technology, Okayama University, Okayama 700-8530, Japan

**Keywords:** robot, CT-guided intervention, needle insertion, prospective, randomized, open-label, blinded-endpoint trial

## Abstract

**Background:**

Robotic needle insertion under CT guidance has been developed, but data on comparison with manual insertion are still lacking.

**Purpose:**

To compare robotic versus manual needle insertion for CT fluoroscopy-guided intervention, primarily in terms of insertion accuracy.

**Materials and Methods:**

This was a prospective study between May 2020 and March 2023 at a single site. The cohort comprised 22 patients undergoing CT (Aquilion One or Aquilion CX; Canon Medical Systems) fluoroscopy-guided biopsy, who were randomly allocated to either the robotic or manual group. The robot used (Zerobot; Medicalnet Okayama) is not yet commercially available. A biopsy introducer needle was inserted by 1 of 3 physicians using a remote-control robot in the robotic group, versus by 1 of 3 different physicians by hand in the manual group. The primary endpoint was needle insertion accuracy, which was defined as the 3-dimensional Euclidean distance between a predetermined target point and the needle tip after insertion. The non-inferiority of robotic insertion to manual one was then tested. Adverse events were evaluated. Statistical comparisons were made between the 2 groups.

**Results:**

Technical success and pathological findings were confirmed in all patients of the 2 groups. The mean and SD of needle insertion were 4.8 mm ± 2.6 in the robotic group and 7.0 mm ± 3.1 in the manual group (*P* < .001). The mean difference in accuracy between the 2 groups (robotic minus manual group) was −2.1 mm (95% CI, −4.7 to 0.4). Effective dose to physicians was zero in all cases in the robotic group, while median dose was 1.0 µSv in the manual group (*P* < .001). Dose length product to patients was not significantly different between the 2 groups (*P* = .100). No major adverse events were observed.

**Conclusion:**

Robotic needle insertion was non-inferior to manual insertion in terms of accuracy, while it effectively eliminated radiation exposure to physicians.

**Trial registration number:**

jRCT2062200013

**Abbreviations**

PROBE = prospective randomized open-label blinded-endpoint; DOF = degrees of freedom; DLP = dose length product; ED = effective dose.

**Summary**

Accuracy of robotic needle insertion for CT-guided intervention was non-inferior to that of manual insertion while eliminating radiation exposure to physicians.

**Key Results**

- Technical success and pathological findings were confirmed in all patients in both groups.
- The mean needle insertion accuracy was 4.8 versus 7.0 mm in the robotic versus manual group, respectively (*P* < .001), with a mean robotic minus manual difference of −2.1 mm (95% CI, −4.7 to 0.4).
- Effective dose to physicians was zero in all cases in the robotic group, while median dose was 1.0 µSv in the manual group (*P* < .001).

## Introduction

Needle insertion for CT-guided interventions had been originally performed under conventional CT guidance, which relies on technicians for CT scanning and requires the time-consuming image reconstruction. To overcome these limitations, CT fluoroscopy was introduced.^1^ With this technique, CT scanning can be performed by the physicians themselves, and images are displayed in near real-time, considerably reducing the procedure time.^2–6^ However, a major disadvantage of this system is the possibility of radiation exposure to physicians operating near the CT gantry.^7–11^

The development of a remote-controlled robot capable of needle insertion under CT guidance has been underway.^12^ Preclinical studies and the clinical study showed that robotic needle insertion was feasible and safe.^13–15^ However, comparison between robotic needle insertion and manual one has been poorly investigated. Therefore, this study aimed to compare efficacy and safety of robotic versus manual needle insertion for CT fluoroscopy-guided interventions.

## Materials and methods

This was a single-center, prospective, randomized, open-label, blinded-endpoint (PROBE) trial that took place from May 2020 to March 2023. The trial was approved by the Institutional Review Board and registered in the Japan Registry of Clinical Trials (jRCT2062200013). Written informed consent was obtained from all patients. This study involved an interim evaluation, in-house monitoring, and audit. This trial does not involve participants overlapping with any previous studies.

We also evaluated the robotic insertion of an ablation needle in an exploratory single-arm cohort during the same period. The results of this exploratory cohort are presented in Supplemental Material S3.

### Cohort

**Figure 1.**
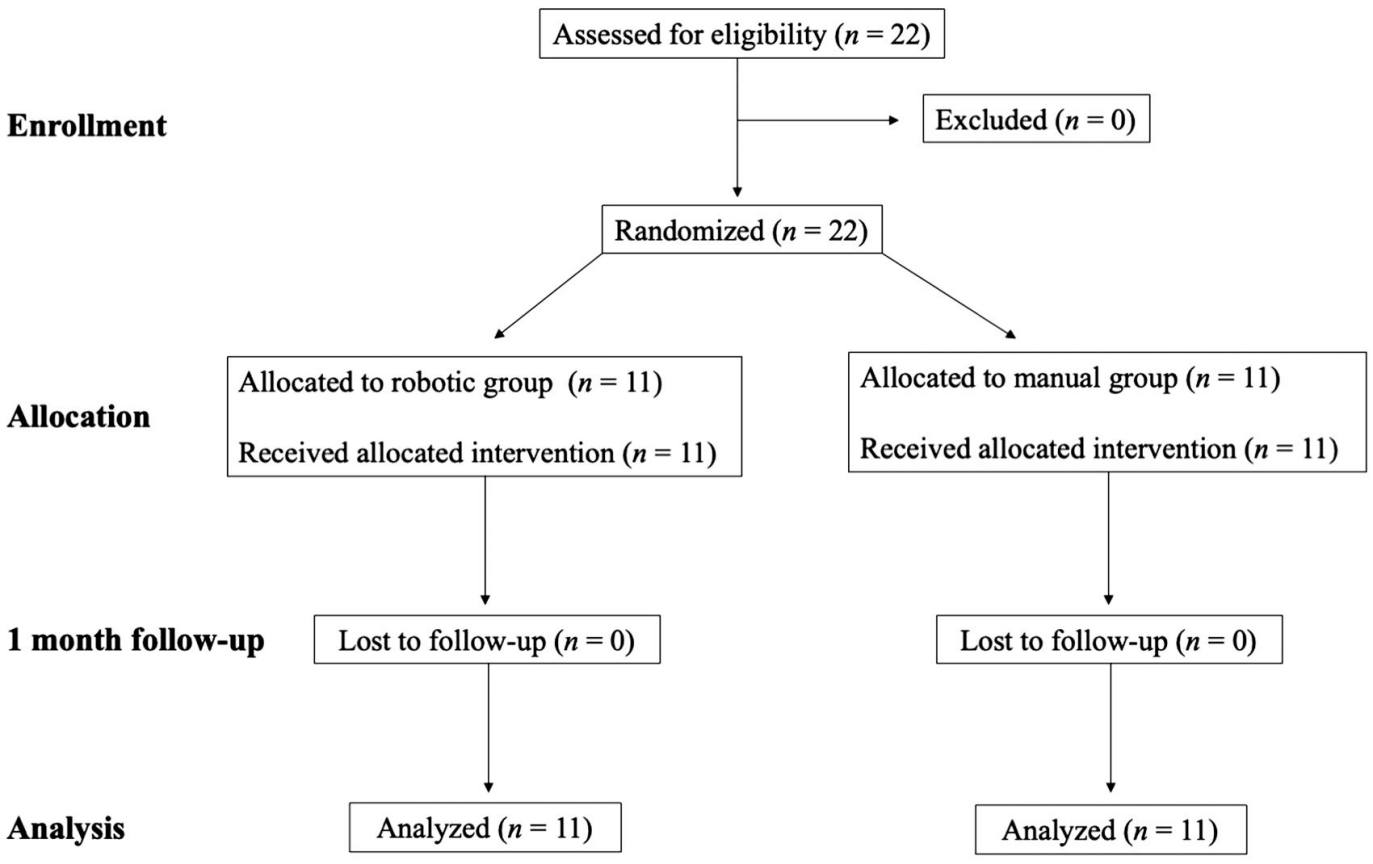
Study flow chart.

Eligibility criteria are shown in Table 1. CONSORT flow diagram is shown in Figure 1. The principal investigator (T.H.) enrolled all patients. The cohort was designed as a PROBE non-inferiority comparative trial, comprising 22 cases that required CT fluoroscopy-guided biopsies in various locations (Table 2). The details of randomization and sample size calculation are shown in Supplemental Material S1.

**Table 1.** Eligibility criteria.

Inclusion criteria
1. Age 20 years or older
2. Platelet count ≥50,000/mm^3^
3. Prothrombin time-international normalized ratio <1.5
4. Single target lesion
5. Lesion in an extremity or the trunk requiring percutaneous biopsy
Exclusion criteria
1. Inability to limit body motion or impaired breath-holding ability
2. Antiplatelet or anticoagulant therapy that cannot be withheld for the procedure
3. At-risk structures (eg, the heart, great vessels, gastrointestinal tract, or pancreas) within 10 mm of the scheduled needle tract
4. Lesion <10 mm in long-axis diameter
5. Scheduled needle tract that passes through bone
6. Pregnant patient
7. Patient who is being enrolled in another trial
8. Patient who is judged unsuitable for any other reason (eg, low compliance) by investigators
9. Lesion in the spinal cord, heart, great vessels, or gastrointestinal tract

**Table 2.** Characteristics in 2 groups.

	Robotic group (*n* = 11)	Manual group (*n* = 11)	*P* value^a^
Age (years)	61.5 ± 11.0	66.7 ± 10.0	.253
Sex			.056
Male	10 (90.9%)	6 (54.5%)	
Female	1 (9.1%)	5 (45.5%)	
Lesion size (mm)	29.4 ± 18.5	24.3 ± 11.1	.444
Lesion location			.587
Lung	4 (36.4%)	6 (54.5%)	
Kidney	3 (27.3%)	2 (18.2%)	
Retroperitoneum	1 (9.1%)	1 (9.1%)	
Pelvis	1 (9.1%)	0 (0%)	
Iliac bone	1 (9.1%)	0 (0%)	
Lower thigh	1 (9.1%)	0 (0%)	
Psoas muscle	0 (0%)	1 (9.1%)	
Liver	0 (0%)	1 (9.1%)	
Needle tract length (mm)	47.7 ± 23.7	60.5 ± 20.4	.189
Patient’s positioning during procedure			.580
Supine	5 (45.5%)	6 (54.5%)	
Prone	5 (45.5%)	5 (45.5%)	
Lateral	1 (9.1%)	0 (0%)	
Number of different physicians	3	3	

Data are expressed as mean ± SD for continuous variables and number (%) for categorical variables.

a Calculated using a 2-sample Student’s *t*-test for continuous variables and a Pearson’s chi-square test for categorical variables.

### Biopsy procedure

Biopsies were performed under conscious sedation in an inpatient setting. A coaxial system consisting of a biopsy introducer needle (TSK guide needle; TSK Laboratory, Tochigi, Japan) and a cutting biopsy needle (STAR CUT; TSK Laboratory) was used. 19- and 17-gauge introducer needles were used in the lungs and other locations, respectively. A sliding gantry CT scanner (Aquilion One or Aquilion CX; Canon Medical Systems, Otawara, Japan) was used for imaging. CT fluoroscopic images were acquired with a tube voltage of 120 kV; currents of 20 and 30 mA in the lung and other locations, respectively; and collimation of 4 mm. The current and collimation were adjusted if necessary.

The patients were placed in the supine, prone, or lateral position. First, CT scan was performed to plan needle insertion. A needle tract (ie, between a skin entry point and a target point) was lined on the axial CT image with 1.0 mm thickness, meaning that needle insertion was performed in-plane. The length and angle of the tract were measured. The CT image with the tract line was recorded using screen capture. After manual administration of local anesthetic, the biopsy introducer needle was robotically or manually inserted toward the target point in the robotic or manual group, respectively (see the sections “Robotic insertion of biopsy introducer needle” and “Manual insertion of biopsy introducer needle,” respectively). Three continuous axial CT fluoroscopic images were observed to check the needle. The needle was inserted while the patients held their breath, when the lesion was affected by respiratory motion. Although the needle angle was adjusted during insertion, repositioning (ie, removing and then reinserting the needle) was not performed. Needle insertion was completed when the physician was satisfied with needle tip position on CT fluoroscopic images. Thereafter, CT scan was performed to evaluate needle insertion accuracy. Subsequently, specimens were manually obtained using the biopsy needle through the introducer needle. After removing the needles, CT scan was repeated to evaluate for adverse events.

#### Robotic insertion of biopsy introducer needle

**Figure 2.**
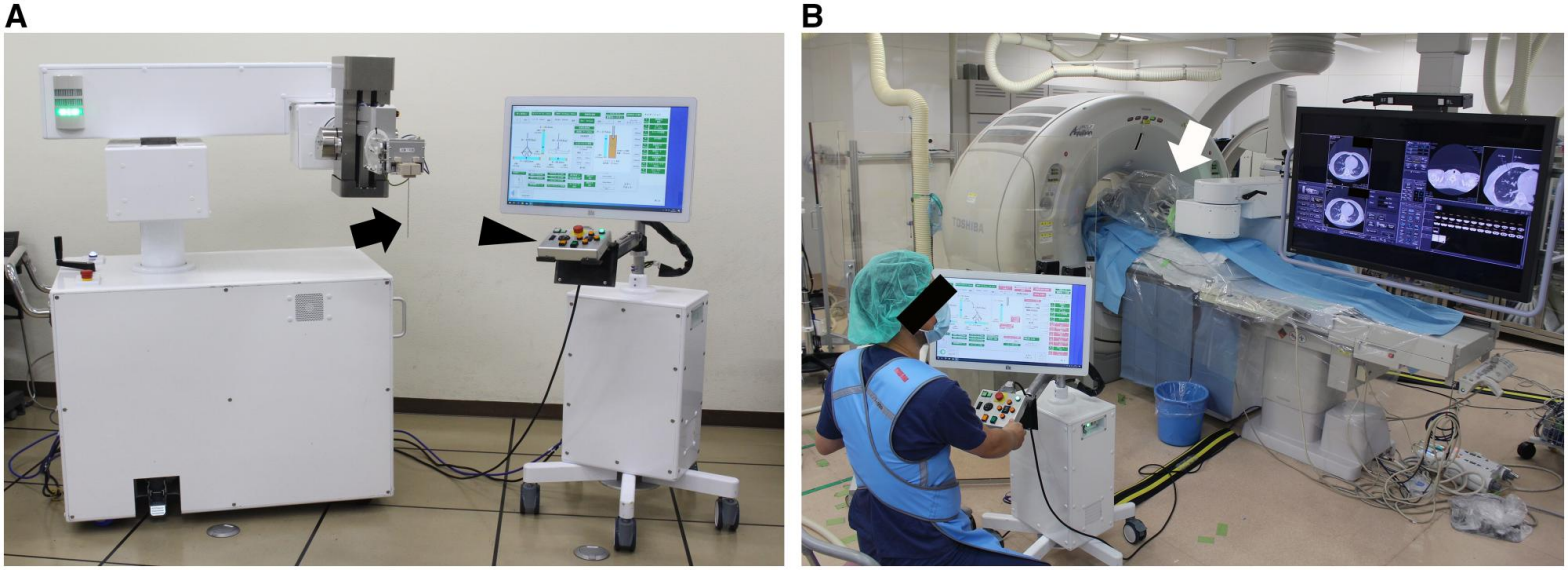
(A) The robotic system consisted of a robot (left) and a user interface (right). The needle (arrow) is attached to the end of the robotic arm. The interface includes a touch panel and a controller (arrowhead). (B) A photograph taken during robotic procedure. The physician sits behind a lead shield a few meters away from the CT gantry and operates the robot (arrow) through the interface under CT fluoroscopic guidance.

The robotic system (Zerobot; Medicalnet Okayama, Okayama, Japan) (Figure 2A) is not yet commercially available, was described in detail in a previous article.^12^ The task of the system is to position, orient, and insert a needle held by the robot, using the physician’s remote operation at the interface. The robot has 6 degrees of freedom (DOF): 2 rotational DOF for needle orientation, 3 linear DOF for needle location, and 1 linear DOF for needle insertion. Each DOF may be driven in either slow or fast mode. Needle insertion speed is 20 mm/s and 5 mm/s in the fast and slow mode, respectively. The robot is positioned beside the CT table for use (Figure 2B).

One of 3 physicians (T.H., Y.M., and T.K.), with 25, 13, and 6 years of experience, respectively, in CT-guided interventions, utilized the robotic system. Before the procedure, they underwent training to operate the robot. A photograph during the robotic procedure is shown in Figure 2B. The detailed techniques of robotic insertion have been described.^15^ Briefly, the physician operated the interface to orient the biopsy introducer needle at a predetermined angle and position the needle tip at the entry point. CT fluoroscopic images were acquired, followed by adjusting the needle as needed. The needle was advanced step-by-step toward the target point. The frequency of CT fluoroscopy usage (intermittently or continuously) during insertion depended on the physician.

#### Manual insertion of biopsy introducer needle

Manual insertion was performed by 1 of 3 different physicians, 2 (K.T. and M.U.) of whom possessed 12 years of experience in CT-guided interventions, while the third (S.K.) had 7 years of experience. A navigation device or planning software was not used.

The physician held the biopsy introducer needle using a pair of plastic forceps and set the needle tip at the entry point. CT fluoroscopic images were acquired, followed by needle adjustment as needed. The needle was manually advanced toward the target point. Unlike the robotic group, all physicians used CT fluoroscopy intermittently to confirm and adjust the needle and not continuously during needle advancement.

### Endpoints

#### Primary endpoint

**Figure 3.**
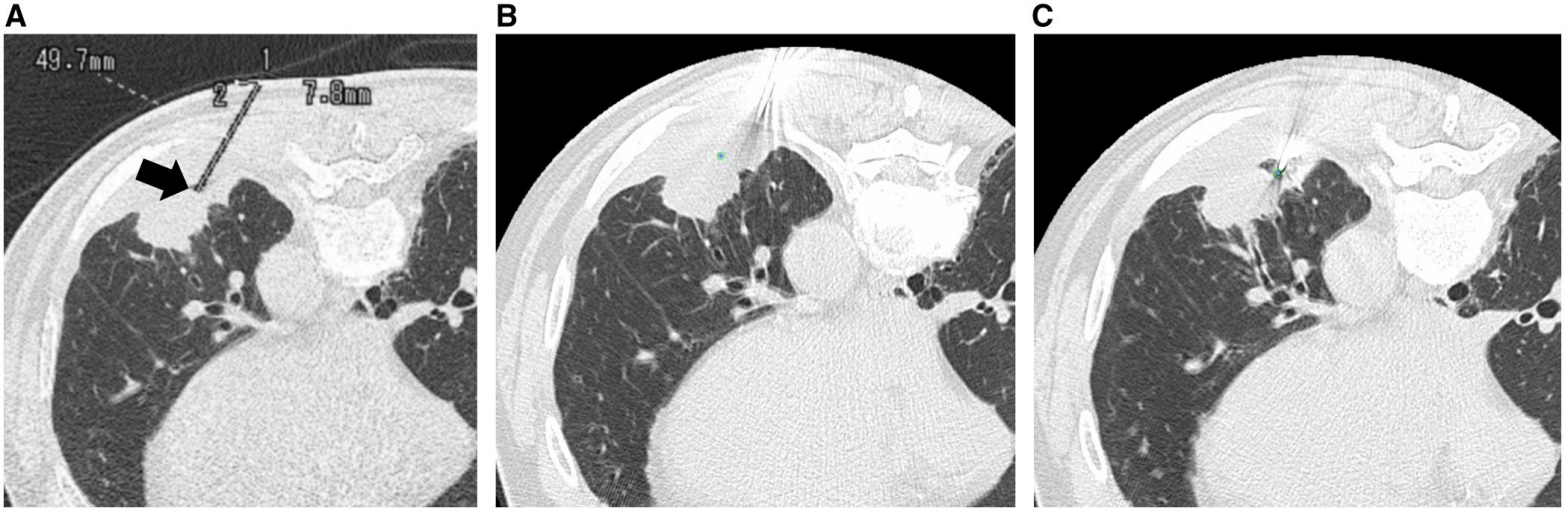
(A) Screen capture of the axial CT image (1 mm thickness) before needle insertion showing the planned needle tract line. The distal end of the needle tract line (arrow) represents the target point. (B) Axial CT image (1 mm thickness) after needle insertion. The target point (circle) is defined based on the screen capture of the axial CT image before insertion (A). The CT coordinates of the target point are *X*: 25.91 mm, *Y*: −8.96 mm, *Z*: −1688.4 mm. (C) Axial CT image (1 mm thickness) after needle insertion. The needle tip (circle) is defined. CT coordinates of the needle tip are *X*: 18.58 mm, *Y*: −11.7 mm, *Z*: −1684.4 mm. The accuracy of the needle insertion (ie, 3-dimensional Euclidean distance between the target point and the needle tip) was calculated to be 8.8 mm.

The primary endpoint was needle insertion accuracy, which was defined as the distance between the predetermined target point and the position of the biopsy introducer needle tip after insertion. The detailed evaluation methods of needle insertion accuracy are shown in Supplemental Material S2 and Figure 3.

#### Secondary endpoint

The secondary effectiveness endpoints were technical success (defined as successful insertion of the needle tip into the lesion), pathological results, dose length product (DLP) to patients and effective dose (ED) to physicians during needle insertion, CT fluoroscopy time (ie, duration of use of CT fluoroscopy) during needle insertion, and needle insertion time (ie, time between the initial and the last CT fluoroscopic scan). ED to physicians was measured with an electronic dosimeter (Hitachi-Aloka Medical, Tokyo, Japan) on the upper chest outside the lead apron. Technical success was evaluated by the central evaluation committee.

#### Safety endpoint

The safety endpoint was adverse events, which were graded according to the Society of Interventional Radiology Clinical Practice Guidelines.^16^

### Statistical analyses

Data were expressed as mean ± SD for normally distributed data and median and interquartile range for non-normally distributed data. To evaluate the non-inferiority of robotic insertion compared to manual insertion, the mean difference in accuracy between the 2 groups (robotic minus manual group) was calculated, along with the 95% CI. Non-inferiority was considered established if the upper limit of the 95% CI was less than 3.0 mm.

Moreover, the following additional analyses were specifically conducted for this article. Needle insertion accuracy was compared between the 2 groups using a 2-sample non-inferiority Student’s *t*-test with a non-inferiority margin of 3.0. Other continuous values were compared between the 2 groups by using a 2-sample Student’s *t*-test for normally distributed data and a Wilcoxon rank-sum test for non-normally distributed data. Categorical values were compared with a Pearson’s chi-square test.

A *P* value <.05 was considered statistically significant. Statistical analysis was performed by using software SAS version 9.4 (SAS Institute, Cary, North Carolina). Clinical research data management and statistical analyses were managed by data managers and independent statisticians (M.Y. and K.S.), respectively.

## Results

### Characteristics of population

The characteristics in the 2 groups are summarized in Table 2. There was no significant difference in age, sex, lesion size, lesion location, needle tract length, or patients’ positioning during the procedure between the 2 groups.

### Primary endpoint

**Figure 4.**
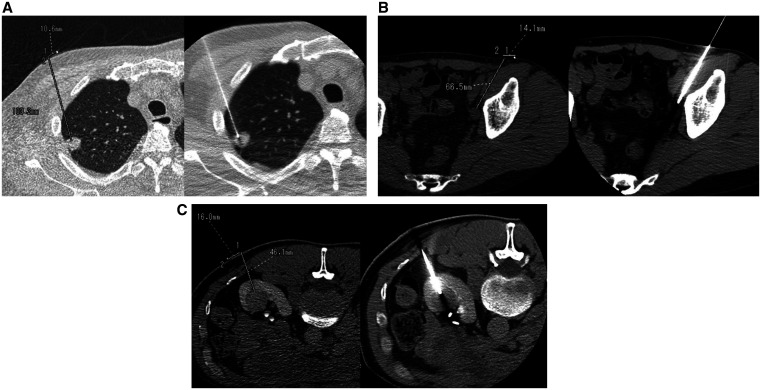
Robotic needle insertion at different anatomic sites (A: lung, B: pelvis, C: kidney). The left images show the planned needle tract, while the right images show the needle after insertion.

Three cases of robotic insertion are shown in Figure 4. Mean insertion accuracy was 4.8 mm ± 2.6 (SD) in the robotic group and 7.0 mm ± 3.1 (SD) in the manual group (*P* < .001) (Table 3). The mean difference of accuracies between the 2 groups (robotic minus manual) was −2.1 mm (95% CI, −4.7 to 0.4).

**Table 3.** Results of effectiveness endpoints.

	Robotic group (*n* = 11)	Manual group (*n* = 11)	*P* value
Needle insertion accuracy (mm)	4.8 ± 2.6^a^	7.0 ± 3.1^a^	<.001^c^
Technical success	11 (100%)	11 (100%)	
Effective dose to physicians during needle insertion (µSv)	0.0 (0.0-0.0)^b^	1.0 (1.0-2.0)^b^	<.001^d^
Dose length product to patients during needle insertion (mGy · cm)	46.4 (26.4-97.3)^b^	32.0 (19.2-38.4)^b^	.100^d^
CT fluoroscopy time during needle insertion (s)	21.4 ± 19.2^a^	8.8 ± 4.9^a^	.048^e^
Needle insertion time (min)	5.1 (3.3-9.0)^b^	4.1 (3.3-5.3)^b^	.212^d^
Pathological result (*n* = 11 in each group)			.395
Adenocarcinoma	2 (18.2%)	4 (36.4%)	
Clear cell carcinoma	2 (18.2%)	2 (18.2%)	
Fibrous tissue	2 (18.2%)	1 (9.1%)	
Squamous cell carcinoma	2 (18.2%)	0 (0%)	
Neurogenic tumor	2 (18.2%)	1 (9.1%)	
Metastatic urothelial carcinoma	0 (0%)	2 (18.2%)	
Metastatic bone tumor	1 (9.1%)	0 (0%)	
Small cell carcinoma	0 (0%)	1 (9.1%)	

a Mean ± SD.

b Median (Q1-Q3).

c Calculated using a 2-sample non-inferiority Student’s *t*-test with a non-inferiority margin of 3.0.

d Calculated using a Wilcoxon rank-sum test.

e Calculated using a 2-sample Student’s *t*-test.

### Secondary endpoint

Technical success and pathological findings were confirmed in all patients of the 2 groups (Table 3). In the robotic group, the ED to physicians during needle insertion was zero in all cases, compared with median 1.0 µSv in the manual group (*P* < .001) (Table 3). The DLP to patients was not significantly different between the 2 groups (*P* = .100); however, CT fluoroscopy time was significantly longer in the robotic group (*P* = .048) (Table 3). Needle insertion time was not significantly different between the 2 groups (*P* = .212) (Table 3).

### Safety endpoint

The robotic group experienced 12 minor (grade A or B) events in 7 patients, while the manual group experienced 20 minor events in 9 patients (Table 4). No major adverse events (ie, grades C-F) or events related to the use of the robot were observed. The incidence of patients who developed events was not statistically different between the 2 groups (*P* = .338).

**Table 4.** Adverse events.

Grade^a^	Robotic group	Manual group
A	Bleeding (*n* = 7)	Bleeding (*n* = 10)
	Pneumothorax (*n* = 2)	Pneumothorax (*n* = 1)
	Pain (*n* = 1)	Pain (*n* = 5)
	Fever (*n* = 1)	Vagal reflex (*n* = 1)
B	Skin papilloma^b^ (*n* = 1)	Bleeding (*n* = 2)
		Pain (*n* = 1)

a Grade according to the Society of Interventional Radiology Clinical Practice Guidelines A = minor, no therapy or consequences; B = minor, nominal therapy and no consequences.

b The event was considered unrelated to the procedure.

## Discussion

This study demonstrated that robotic needle insertion was clinically successful, as all robotic cases achieved both technical success and pathological results. With regard to needle insertion accuracy, the non-inferiority of robotic insertion to manual one in terms of accuracy was successfully proven. Physicians were not exposed to radiation during robotic insertion in this study, whereas previous studies on manual CT fluoroscopy-guided biopsy reported a mean estimated ED of 0.054 mSv^9^ and an equivalent dose of 4.7 µSv.^10^ Therefore, the clinical implications of this study suggest that the use of the robot may enable clinical success without compromising insertion accuracy or exposing physicians to radiation.

Robots for CT-guided needle insertion fall into 2 categories. The first type assists needle insertion by robotically targeting a needle guider^17–24^; however, needle insertion must still be performed manually through the guider. These navigation robots were shown to increase needle insertion accuracy^20^ and reduce procedure time and radiation exposure to patients.^24^ The second type of robot boasts fully robotic needle insertion capabilities.^12–15^^,^^25–28^ Among such needle insertion robots, the Acubot has been the only one to be evaluated in a randomized trial comparing robotic and manual insertion.^26^ This trial demonstrated several advantages of robotic insertion, including fewer needle adjustments, shorter procedure time, and less radiation exposure for both physicians and patients. Regarding the robot used in this study, a phantom study^13^ demonstrated that inserting biopsy introducer needles using the robot under CT fluoroscopic guidance prevented radiation exposure to physicians, with an insertion accuracy equivalent to manual insertion. Subsequent experiments on swine showed that the robotic insertion of biopsy introducer needles was safe and accurate at several anatomical locations.^13^ Accurate robotic insertion was later confirmed using various ablation needles in swine.^14^ In 2018, the first clinical feasibility study^15^ was conducted using 10 patients undergoing CT fluoroscopy-guided biopsy. Robotic insertion of a biopsy introducer needle was confirmed to be both safe and successful in various locations, including the lungs, mediastinum, and kidneys.^15^ Then, we conducted this randomized trial comparing robotic and manual insertions.

Compared with manual insertion without the use of navigation device, advantages of robotic insertion include accurate needle targeting before insertion and needle stability during insertion. Such advantages seemed to contribute to the non-inferiority with acceptable safety, despite the quite limited experience in robotic procedures. Recently, 41 patients who underwent CT-guided ablation of abdominal tumors with a navigation robot (EPIONE) for needle insertion were reported.^23^ Seventy-six of 79 (96%) needle insertions were technically successful; 2 required complete manual reinsertion, while one required major needle adjustments. The mean lateral distance between the needle tip and planned trajectory was 3.2 ± 4.5 (SD) mm before adjustments, and the mean 3-dimensional distance was 1.6 ± 2.6 (SD) mm after manual depth and lateral adjustments. Then, one might suggest that the accuracy of robotic needle insertion in this study was not high. Given that the target point was a relative position rather than an absolute one in relation to the lesion and other nearby tissues, it often shifted during insertion. On CT fluoroscopic images, the physician had to recognize this shift and adjust the needle angle to follow the target point. The most challenging aspect was the inability of the physician to precisely identify the exact target point on the CT fluoroscopic images during needle insertion. Hence, we believe that the deviations in needle insertion were not due to errors by the robotic system, but rather to perception errors by the physicians when identifying the target point.

Robotic insertion was associated with the extended use of CT fluoroscopy. While CT fluoroscopy was intermittently used during manual procedures, it was often used continuously during robotic procedures. This was mainly because of the unfamiliarity with the robotic procedure, which prompted the physicians to use CT fluoroscopy more frequently to confirm needle insertion. Furthermore, the lack of radiation exposure to physicians during robotic procedures may have been another enabling factor for the increased CT fluoroscopy usage.

While the robotic system shows promise, further development and evaluation could enhance its capabilities. Currently, physicians manually operate the robot at the interface. However, the potential for automating robotic procedures to some extent could offer considerable advantages, such as decreasing both procedure time and radiation exposure to patients. Additionally, the introduction of a breath-synchronization system could further facilitate robotic insertions. Moreover, the applicability of this robotic system in telemedicine could be investigated in future studies.

This trial faced various limitations. Besides limited generalizability due to the small population and being performed in a single institution, the physicians could not be blinded to the procedure type (robotic or manual). This might have introduced a potential bias during needle insertion. The physicians in the 2 groups were different, which could have led to an operator bias. Furthermore, there was a bias in physicians’ experience between the 2 procedures, which might have resulted in a bit longer needle insertion time in the robotic group. Moreover, the physicians’ learning curve with the robot might have affected robotic needle insertion time. Given that the lesion site may have influenced needle insertion (eg, by respiratory motion and organ stiffness), stratification by both lesion size and site would have been preferable. However, the cohort size was too small to allow for stratification by site. Consequently, lesion site distribution was biased between the 2 groups; for example, more patients with a static target (eg, lesions in the bone and thigh) were included in the robotic group. Navigation device or planning software, used by some proceduralists, was not used in the manual insertion arm. The use of CT fluoroscopy during needle insertion was not uniform in the 2 groups. Additionally, because this study aimed to demonstrate the non-inferiority of robotic insertion, the superiority of robotic insertion over manual one was not demonstrated.

In summary, robotic needle insertion proved non-inferior to manual insertion in terms of accuracy and freed physicians from radiation exposure. The accurate and stable needle posture offered by the robot may enable accurate insertion even by novices in CT-guided interventions, as well as accurate out-of-axial CT plane insertion. These remain areas for future research.

## Supplementary Material

umaf010_Supplementary_Data

## Data Availability

Data analyzed during the study were provided by a third party. Requests for data should be directed to the provider indicated in the Acknowledgments.
