# Supplementary material for: Comparison of robotic versus manual needle insertion for CT-guided intervention: prospective randomized trial
Source: Radiol Adv. 2025 Apr 4;2(2):umaf010. doi: 10.1093/radadv/umaf010 (PMC12429234; doi:10.1093/radadv/umaf010)
Supplement: umaf010_Supplementary_Data [file umaf010_Supplementary_Data.zip › Supplemental material (Revision).pdf]

Supplemental Material 1.

The details of randomization and sample size calculation

The cases were randomly allocated to either the robotic or manual group by the independent data center. Randomization was done using permuted blocks with randomly varying block sizes and stratified by lesion size. Only data managers from third-party contract research organizations were able to access the information about randomized assignment before database lock. The cohort size was determined based on the following statistical consideration. The difference in mean needle insertion accuracy between the two groups and common standard deviation (SD) in both groups were assumed as 0 mm and 2.3 mm, respectively. The non-inferiority margin was set at 3 mm. In clinical settings, needle insertion is typically guided by CT images with 4- to 5-mm section thickness. Thererby, detecting needle deviations of just a few millimeters in the craniocaudal direction can be challenging. The margin of 3 mm was established to ensure that such deviations are considered acceptable. Then, the sample size of each group was determined as 11 cases to keep a power of 80% under a significant level of 0.05 (two-sided).

Supplemental Material 2.

The detailed evaluation methods of needle insertion accuracy

Needle insertion accuracy was evaluated on axial CT images (1.0 mm thickness, 0.8 mm intervals) obtained immediately after insertion using software (OsiriX MD, version 11.0.4, Pixmeo SARL). With reference to the screen capture of the CT image before insertion (Figure 3A), the target point was determined on the CT image (Figure 3B). The target point was not an absolute position, but rather relative position with respect to the lesion and other nearby tissues. Therefore, the target point after insertion often shifted from its original position (i.e., the position before insertion) due to factors such as tissue displacement and deformation, and discrepancies in breath-holding timing. The needle tip was also identified on the CT image (Figure 3C). The three-dimensional Euclidean distance between the target point and needle tip

was calculated using their CT coordinates (Figure 3B, C). Three interventional radiologists from the independent central evaluation committee evaluated the accuracy while blinded to the allocated group in each case. The mean of the needle insertion accuracy values that were calculated by the three evaluators was adopted as a representative in each case.

Supplemental Material 3.

The trial included another exploratory cohort, which was designed as a single-arm study aimed at evaluating robotic insertion of an ablation needle rather than comparison with manual insertion. The eligibility criteria of this cohort are shown in Supplemental Table 1. The cohort intended to include five cases undergoing CT fluoroscopy-guided ablation for hepatic cancer or renal cell carcinoma (RCC). However, ultimately, no case of hepatic ablation was enrolled, and all patients underwent cryoablation (CRA) for RCC. All patients were male, with a mean age of 73.4 years (range, 63–84 years). The mean size of RCC was 27.2 mm (range, 14–40 mm).

The effectiveness endpoints included needle insertion accuracy, technical success (defined as complete circumscription of the tumor by the ablation zone on CT or MRI after two days), technique effectiveness (defined as no residual unablated part of the tumor on CT or MRI at 1 month), DLP to patients and ED to physicians during robotic insertion, CT fluoroscopy time during robotic insertion, and needle insertion time. Technical success and technique effectiveness were evaluated by the central evaluation committee. Descriptive statistics were used to evaluate other effectiveness endpoints. Adverse events were also evaluated as the safety endpoint.

Prior to CRA, superselective transarterial embolization was performed in a separate session, using iodized oil and a gelatin sponge. This was expected to improve visualization of RCC on CT fluoroscopic images during CRA by deposition of radiopaque iodized oil, and to reduce the cold sink effect and the potential risks of bleeding.

Renal CRA was performed with the patients in a prone position under local anesthesia and conscious sedation. An argon-helium CRA system (Visual-ICE; Boston Scientific, Marlborough, MA) with 17-gauge cryoneedles (IceRod or IceSphere; Boston Scientific) was used. The number and type of cryoneedles used were determined by the consensus of experienced interventional radiologists in a conference. Consequently, three cryoneedles were used in all cases. CT fluoroscopic images were acquired with a tube voltage of 120 kV, a current of 30 mA, and a collimation of 4 mm.

One of two trained physicians (T.H., Y.M.) operated the robot in each case. In all cases, the first cryoneedle was inserted using the robot; the insertion technique was quite similar to that used in the text. Then, CT scan was performed to evaluate insertion accuracy. Subsequent two cryoneedles were inserted manually to ensure that the distance among the cryoneedles was less than 1.5 cm. Ablation protocol included two 15-minute freezing cycles separated by two or more minutes of passive thawing. CT scan was performed at the end of the freezing period to assess the ablation zone. Clinical endpoint of ablation was defined as coverage of the RCC with an adequate ($\geq$ 6 mm) margin by the ablation zone. After the removal of the cryoneedles, CT scan was repeated to evaluate adverse events.

Patients underwent dynamic CT or MRI to evaluate technical success on postprocedural day 2. Patients visited the hospital for follow-up at 1 month, with undergoing additional dynamic CT or MRI to evaluate technique effectiveness.

Technical success and technique effectiveness were confirmed in all patients. The results of other effectiveness endpoints are shown in supplemental Table 2. No major adverse events or events related to the use of the robot were observed.

Robotic insertion accuracy of the cryoneedle seemed comparable to that of the biopsy introducer needle, while insertion of the former seemed to be associated with increased radiation exposure to patients, CT fluoroscopy time, and insertion time. These results suggest that needle insertion may be more challenging in the kidney because of its movable nature,

requiring more needle adjustments during insertion. In this cohort, only the first cryoneedle was robotically inserted and subsequent cryoneedles were inserted manually to avoid potential interference between the robotic arm and the already-placed initial cryoneedle, which highlights a limitation of the robotic system.

Supplemental Table 1. Eligibility Criteria

Inclusion criteria
1) Age 20 years or older
2) Platelet count ≥ 50,000/mm^3
3) Prothrombin time-international normalized ratio < 1.5
4) Single target lesion
5) Hepatic or renal cancer that requires percutaneous ablation and is unsuitable for ultrasound-guided ablation

Exclusion criteria
1) Inability to limit body motion or impaired breath-holding ability
2) Antiplatelet or anticoagulant therapy that cannot be withheld for the procedure
3) At-risk structures (e.g., the heart, great vessels, gastrointestinal tract, or pancreas) within 10 mm of the scheduled needle tract
4) Lesion <10 mm in long-axis diameter
5) Scheduled needle tract that passes through bone
6) Pregnant patient
7) Patient who is being enrolled in another trial
8) Patient who is judged unsuitable for any other reason (e.g., low compliance) by investigators
9) Child-Pugh class C
10) Massive ascites
11) Uncontrollable malignancy in locations other than target organ
12) Implantable cardiac pacemaker or implantable cardioverter defibrillator
13) Scheduled other therapies for target lesion within 1 month after ablation
14) Inapplicable contrast-enhanced CT (e.g., allergy for iodine)

Supplemental Table 2. Results of Effectiveness Endpoints

Needle insertion accuracy (mm)	4.7 ± 2.4[a]
Effective dose to physicians during needle insertion (μSv)	0.0 (0.0–0.0)[b]
Dose length product to patients during needle insertion (mGy・cm)	109.8 (109.2–146.6)[b]
CT fluoroscopy time during needle insertion (s)	49.0 ± 11.4[a]
Needle insertion time (min)	9.0 (8.5–9.5)[b]

[a]Mean ± standard deviation

[b]Median (Q1–Q3)
